# Subcutaneous BoNT/A Injection for Intractable Pain and Disability in Complex Regional Pain Syndrome: A Case Report

**DOI:** 10.3390/toxins14060411

**Published:** 2022-06-16

**Authors:** Yan Tereshko, Chiara Dalla Torre, Christian Lettieri, Enrico Belgrado, Gian Luigi Gigli, Mariarosaria Valente

**Affiliations:** 1 Clinical Neurology Unit, Udine University Hospital, Piazzale Santa Maria della Misericordia 15, 33100 Udine, Italy; gianluigi.gigli@uniud.it (G.L.G.); mariarosaria.valente@uniud.it (M.V.); 2 Neurology Unit, Udine University Hospital, Piazzale Santa Maria della Misericordia 15, 33100 Udine, Italy; chiara.dallatorre@asufc.sanita.fvg.it (C.D.T.); christian.lettieri@asufc.sanita.fvg.it (C.L.); enrico.belgrado@asufc.sanita.fvg.it (E.B.)

**Keywords:** CRPS, subcutaneous injection, botulinum toxin, pain, disability

## Abstract

We treated a 51-year-old woman with refractory Complex Regional Pain Syndrome type I (CRPS-I) involving her left hand and forearm with subcutaneous injections of BoNT/A. The injections were performed every 3 months, with a total of six treatments. Each treatment was able to effectively improve pain and motor impairment; however, the duration of the effect was limited to only a few months. BoNT/A could improve patients’ quality of life with CRPS; however, extensive clinical studies are needed to determine its role in clinical practice.

## 1. Introduction

Complex Regional Pain Syndrome (CRPS) is characterized by chronic regional pain, typically distal and disproportionate to the degree of the noxious insult, associated with abnormal skin tropism, motor, sensory, and vasomotor findings of the affected limb on physical examination [1]. Frequent relapses and the lack of an effective therapy lead to impaired quality of life and persistent disability in the affected patients.

In patients with CPRS-I, it is not possible to demonstrate a nerve injury, whereas patients with CRPS-II have a specific nerve injury identified. Although the exact pathophysiology of the syndrome is not known, many findings suggest the involvement of small nerve fibers (A-delta and C fibers). Quantitative sensory testing (QST), laser-evoked potentials (LEP), and skin biopsy are frequently altered in these patients [2,3].

Botulinum toxin type A (BoNT/A) has been demonstrated to be effective in improving pain in many different neuropathic pain syndromes refractory to conventional treatment [4] even though chronic migraine pain is the only approved indication in such contexts [5]; in other cases of neuropathic pain, BoNT/A is suggested as a third-line therapy [6].

In the context of CRPS, very few patients have been treated with BoNT/A, with different approaches such as intramuscular injections [7,8], intra-articular injections [9], and lumbar sympathetic blocks [10], with some degree of improvement in pain. Other studies reported mixed results with subcutaneous injections of BoNT/A [11,12,13]. Here, we report a single case of CRPS-I with almost complete motor impairment and intractable pain successfully treated with subcutaneous injections of BoNT/A.

### Case Report

In 2016, a 51-year-old woman came to the neurologist with a complaint of severe burning, stabbing, and persistent pain in her left hand, associated with intermittent swelling, tingling, hyperhidrosis, and motor dysfunction; cold temperature triggered or exacerbated the symptoms.

Her medical history included chronic migraine and temporal lobe pharmaco-resistant epilepsy. No abnormalities were seen on an MRI of the brain. Her past medication history included gabapentin, carbamazepine, oxcarbazepine, sodium valproate, lacosamide, and levetiracetam. Chronic migraine developed before the onset of CRPS and was treated with flunarizine, amitriptyline, topiramate, and bilateral great occipital nerve anesthetic blocks, with only partial benefit. Three years before the CRPS symptoms, rounds of botulinum toxin therapy permitted optimal control of the migraine. 

Her epilepsy therapy remained unchanged in the prior three years: levetiracetam 1000 mg BID, lacosamide 200 mg BID, and topiramate 50 mg BID.

The patient was diagnosed with CRPS-I according to the Budapest criteria [14] and started therapy with 1 mg/kg of prednisone for 2 months, with subsequent tapering and 300 mg/die of pregabalin with poor benefit. Conventional pain medications (FANS, lidocaine patches, duloxetine, and opioids) were also ineffective. In 2016, she performed three anesthetic blocks with lidocaine of the left ulnar nerve, with pain relief lasting only one week; at the end of 2016, she was treated with two courses of pulsed radiofrequency of the left ulnar nerve, with pain improvement that lasted one month after each attempt.

During 2016 and 2017, she performed physiotherapy cycles without significant improvement, and, in the last months of 2017, she was treated with two anesthetic brachial plexus blocks with partial control of the pain that lasted until the end of 2019; unfortunately, these blocks triggered a seizure immediately and the patient refused to perform the procedure again. In the first months of 2020, the pain worsened progressively, and we decided to treat the patient with subcutaneous injections of botulinum toxin, considering the evidence of its efficacy in neuropathic pain [4].

Upon a neurological examination, the left hand was cold, cyanotic, and swollen with hypoesthesia and allodynia, predominantly in the territory of the ulnar nerve in the hand and of the medial antebrachial nerve in the forearm. She denied any trauma in the previous months and organic causes were excluded.

**Figure 1:**
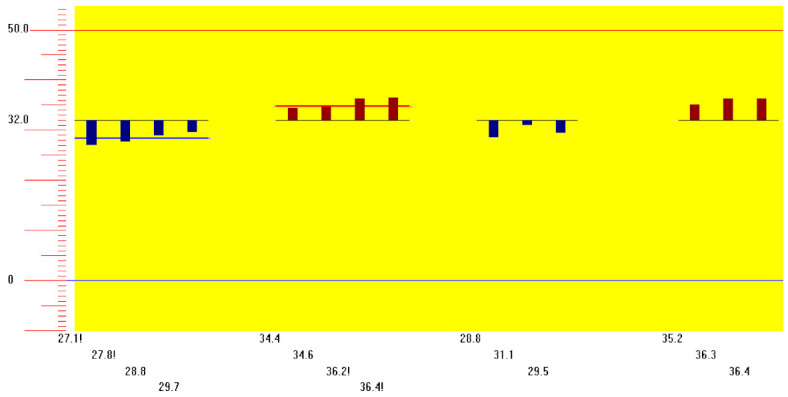
Warm and cold sensation thresholds, in our patient, showed mild small-fibers impairment while cold and heat pain thresholds were significantly lowered, consistently with thermal hyperalgesia. The vertical axis shows temperature (°C). The horizontal axis shows the temperature at which the patient refers to, from the left to the right, cold sensation (blue), warm sensation (red), cold pain (blue), and heat pain (red); the exclamation point “!” means the temperature sensation is over the normality threshold. The mean value for the cold sensation threshold: 28.3 °C; The mean value for the warm sensation threshold: 35.4 °C. The mean value for cold pain: 29.8 °C; The mean value for heat pain: 35.9 °C.

**Figure 2:**
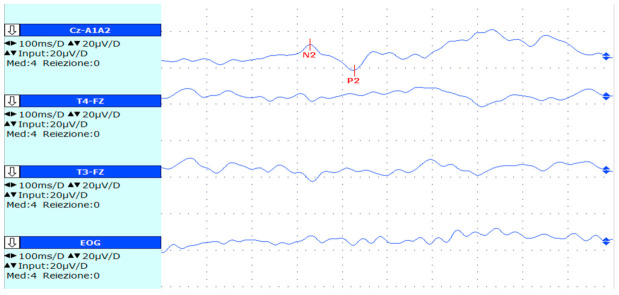
Left hand averaged LEP of our patient: latency of N2 = 329 m; latency of P2 = 427 ms. The N2-P2 vertex complex (which is the most common waveform complex used to assess LEP) is clearly altered (latency normative values for solid-state lasers [15]: N2 < 252 ms; P2 < 415 ms) and detectable from the background noise.

Thermal quantitative sensory testing (QST) and laser-evoked potentials (LEP) were altered in the affected limb (see Figure 1 and Figure 2).

**Figure 3:**
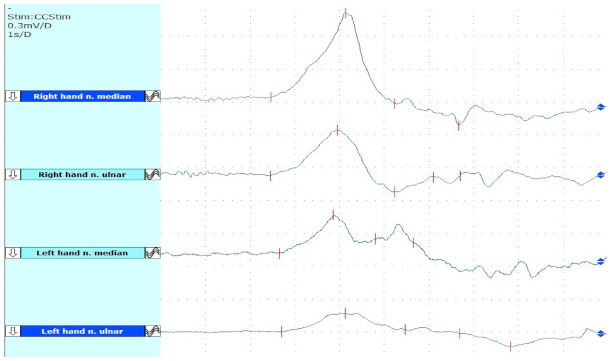
Electric SSR from upper limbs of our patient; this test evaluates the integrity of the sudomotor cholinergic postganglionic fibers. The test is pathological when there is an absent response or when there are clear asymmetries [16]. From top to bottom: the first two traces show right-hand responses. After median nerve stimulation: latency = 2459 ms; amplitude = 1.03 mV; after ulnar nerve stimulation: latency = 2452 ms; amplitude = 0.43 mV. The last two traces show left-hand responses. After median nerve stimulation: latency = 2649 ms; amplitude = 0.26 mV. After ulnar nerve stimulation: latency = 2710 ms; amplitude = 0.19 mV. The response is present bilaterally but the traces in the left hand are slightly prolonged and the amplitudes are reduced in comparison with the right hand.

Nerve conduction studies were normal but sympathetic skin response was abnormal in her left hand (amplitude was reduced by more than 60% in comparison to the healthy side; see Figure 3). An ulnar nerve anesthetic block (1 cc of bupivacaine 2.5 mg/mL) brought significant pain relief along with trophic skin abnormality improvement for a week.

## 2. Results

Improvement of pain and limb motor impairment occurred after one week in all six treatments of BONT/A injections. After the first treatment, the duration of the benefit lasted about 20 days while, after the following injections, it lasted for about two and a half months, with the greatest benefit in the first month and gradual loss of benefit. From the second injection, and in the following treatments, pain and allodynia were greatly reduced as well as the motor impairment of her left limb; in fact, during the first month, the patient was able to carry out almost all the activities of daily life that she used to do before the disease and the only limitations she had were in gardening and repetitive strain activities. Only moderate to severe exposure to cold and intense pressure could provoke her typical pain, although in reduced severity; spontaneous pain was absent and abnormal sensation was greatly reduced or absent. During the second month, the improvement started to slowly fade with a gradual reappearance/worsening of allodynia, trophic abnormalities, and pain. No side effects were reported. Three months after each treatment, the pain and the limb disability returned to the baseline.

The details of clinical scale scores before and after each treatment are reported in Table 1.

## 3. Discussion

Few reports are available regarding the use of subcutaneous BoNT/A in patients with CRPS.

In 2010 [11], an interesting randomized double-blind, placebo-controlled crossover with 8 (out of 14) CRPS patients treated with subcutaneous and intradermal injections of BoNT/A did not show significant improvement regarding allodynia. However, this study was conducted in advanced forms of CRPS, the injections were intolerable, and the sites of the injections and the limb they treated were not reported.

A case report in 2012 [12] demonstrated an improvement in joint range of movement and pain in a patient with CRPS-I after subcutaneous injection of BoNT/A in the dorsum of her hand.

In 2020 [13], a CRPS-II patient was treated with subcutaneous injections of BoNT/A in the palm with significant pain improvement.

Our patient was treated in the left hand, both palm and dorsum, and in the ventral aspect of the forearm, with effective control over pain and allodynia; the treatment was effective in improving motor impairment as well, as assessed with the DASH score. The patient was satisfied with the treatment (see PGIC, Table 1) and was able to conduct most of her daily activities after each treatment. Spontaneous pain was almost abolished and provoked pain occurred only after moderate–severe cold exposure, but the intensity was mild.

The duration and the intensity of the effect of BoNT/A seem to be related to the dose but also the sites of injection. The dose, dilution, and injected volume are important factors for the local spreading of botulinum toxin and it is suggested to dilute the botulinum toxin according to the area of interest [17,18]. BoNT/A dilution in a higher volume of saline determines a higher fluid load and probably a larger spreading remote from the site of inoculation and reduces the unit loss in the hub of the needle [17,19].

The thinly unmyelinated fibers mainly involved in CRPS (A-delta and C fibers) have receptive fields that innervate structures outside the traditional dermatomes [20] and their axon terminals can be electrically coupled with adjacent fibers [21], permitting communication between them. In addition, central sensitization in chronic neuropathic pain is involved in the spreading over of mechanical dynamic allodynia and hyperalgesia into the adjacent areas from the primary zone of injury [22]. BoNT/A efficacy could be greater in treating the adjacent nerve territories and not only focusing on the region of allodynia and pain.

BoNT/A exerts its functions on modulating neuropathic pain by inhibiting the release of neurotransmitters involved in peripheric and central sensitization, in particular CGRP and substance P [23,24,25,26], but not the release of GABA [27]. It interferes with the plasma membrane expression of TRPV1 in sensory fibers, the sensory surface of neuronal ganglia, and beyond the ganglia in the central nervous system [28,29], probably due to axonal retrograde transport and trans-synaptic transport [29,30,31]; BoNT/A has proven to enhance the segmental dorsal horn and brainstem endogenous opioid and GABA inhibitory systems [32,33] and attenuate microglia activation [34,35]. Furthermore, BoNT/A has shown selectivity for TRPV1-expressing afferents, such as nociceptive C fibers [36,37], involved in pain and mechanical stimulation but not in other sensory modalities.

In the first round of injections, we treated only the ulnar territory, while in the following rounds, we decided to apply a more widespread approach with an improvement of the efficacy on the pain and disability as well as a longer duration of the benefit; in fact, low doses of BoNT/A are insufficient for the spreading into the central nervous system [36,38]. Regarding the efficacy of BoNT/A in neuropathic pain, a recent meta-analysis of randomized controlled studies reported greater efficacy of BoNT/A in reducing pain than placebo [39]. In the context of CRPS, a meta-analysis of placebo responses in long-standing CRPS patients reported no evidence of placebo response [40], but there were no studies regarding therapy with BoNT/A. The placebo effect could play an important role in CRPS and BoNT/A therapy; however, large multicentric studies are needed to determine its contribution in this setting.

Further research and extensive clinical studies are needed to determine the most efficient injection technique with BoNT/A in patients with CRPS.

## 4. Materials and Methods

The patient gave her informed consent for the treatment with botulinum toxin and for her images and other clinical information to be reported in the journal. The patient did not take any pain medications at the baseline assessment and during the follow-up period; the patient continued her therapy for epilepsy and botulinum toxin for chronic migraine.

Injections of BoNT/A and clinical examination were performed every 3 months.

Pain and disability assessments were performed at the time of the treatment and 1 month later by the means of VAS; disability of the arm, shoulder, and hand (DASH) [41]; and neuropathic pain symptoms inventory (NPSI) [42]. The patients’ global impression of change (PGIC) [43] scale was assessed 1 month after every session.

Treatment was performed with subcutaneous injections of botulinum toxin type A (BOTOX^®^) using a 27-gauge × 12.5 mm needle. Local lidocaine 5% pomade was applied for local anesthesia. For subcutaneous injections, we gently grasped the area of the skin surrounding the injection site to separate it from the muscular layer; then, we thrust the needle, with an injection angle of 30°, into the subcutis. This procedure was easy to perform in the forearm and the dorsum of the hand; in the palm, we treated the areas where the skin had better mobility against the underlying layers and better pinching to avoid intramuscular injections. Ultrasound guidance was not used; it could be possible that a minimum amount of BoNT/A IU reached the deep fascia and then the muscular layer beneath; however, the patient did not develop muscular weakness at the visits that were one month after each round of injections.

In the first session with BoNT/A, a 100 IU vial was diluted with 2 mL of 0.9% sodium chloride; 25 IU were injected in the left hand into the ventral and dorsal aspects of the IV and V fingers at 10 sites (2.5 IU per site) and 25 IU were injected into the ventral aspect of the left forearm, within the territory of the medial antebrachial cutaneous nerve at 5 sites also (5 IU per site).

**Figure 4:**
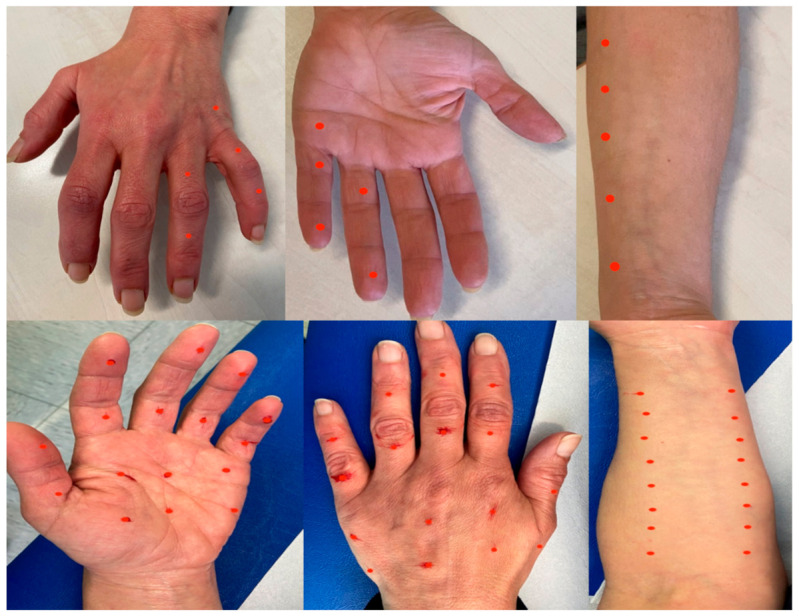
The upper half of the figure shows the sites treated with 50 IU of BoNT/A with 2 mL of 0.9% sodium chloride dilution in the first session; 2.5 IU per site in the hand and 5 IU per site in the forearm. The lower half of the figure shows the sites treated with 80 IU of BoNT/A with 2 mL of 0.9% sodium chloride dilution for the forearm and 4 mL for the hand in the following session; 2.5 IU per site in the forearm and 1.25 IU per site in the hand. The red dots indicate the sites of the injections.

During the following sessions, we decided to modify the dilution with 0.9% sodium chloride to enhance the diffusion in the hand as follows: 2 mL for the forearm and 4 mL for the hand of 0.9% sodium chloride; 40 IU were injected into the ventral aspect of the forearm into the medial and lateral antebrachial nerve territories at 16 sites (2.5 IU per site) and 40 IU were injected into the dorsal and ventral aspects of the hand at 32 sites (1.25 IU per site). The procedures were performed without any side effects and the injection pain was sufficiently tolerated by the patient. The precise sites treated are shown in Figure 4.

## Figures and Tables

**Table 1:** Six treatments were performed. Legend: BoNTA, Botulinum Toxin type A; T0, assessment at the moment of BoNT/A injection; T1, assessment one month after BoNT/A injection; NPSI, neuropathic pain symptom inventory; DASH, disability of the arm, shoulder, and hand; PGIC, patients’ global impression of change.

BoNT/A Injections	Assessment	NPSI	VAS	DASH	PGIC	Duration (Days)	Side Effects
1st Treatment	T0	80	95	96.7%			
	T1	26	60	56.7%	4	20	None
2nd Treatment	T0	75	90	87.5%			
	T1	11	30	28.3%	5	65	None
3rd treatment	T0	67	90	85.8%			
	T1	3	11	7.5%	6	70	None
4th Treatment	T0	62	90	87.5%			
	T1	16	13	15%	6	60	None
5th Treatment	T0	63	87	96.7%			
	T1	8	13	8.3%	6	65	None
6th Treatment	T0	70	89	87.5%			
	T1	9	11	7.5%	6	70	None

## Data Availability

Not applicable.
